# Patient Knowledge and Trust in Health Care. A Theoretical Discussion on the Relationship Between Patients’ Knowledge and Their Trust in Health Care Personnel in High Modernity

**DOI:** 10.1007/s10728-023-00467-7

**Published:** 2023-10-09

**Authors:** Stein Conradsen, Henrik Vardinghus-Nielsen, Helge Skirbekk

**Affiliations:** 1 Department of Education, Faculty of Humanities and Education, Volda University College, Volda, Norway; 2 Department of Health Science and Technology, The Faculty of Medicine, Aalborg University, Aalborg, Denmark; 3 Department of Nursing and Health Promotion, Faculty of Health Sciences, OsloMet, Oslo, Norway

**Keywords:** Knowledge theory, Trust, Dewey, Luhmann, Giddens, Health care

## Abstract

In this paper we aim to discuss a theoretical explanation for the positive relationship between patients’ knowledge and their trust in healthcare personnel. Our approach is based on John Dewey’s notion of continuity. This notion entails that the individual’s experiences are interpreted as interrelated to each other, and that knowledge is related to future experience, not merely a record of the past. Furthermore, we apply Niklas Luhmann’s theory on trust as a way of reducing complexity and enabling action. Anthony Giddens’ description and analysis of the high modern society provides a frame for discussing the preconditions for patient-healthcare personnel interaction. High modernity is dominated by expert systems and demands trust in these. We conclude that patient knowledge and trust in healthcare personnel is related because both knowledge and trust are future- and action-oriented concepts. The traits of high modernity provides opportunities and challenges as the personnel can and must perform discretion. This discretion must be made in a context where knowledge is considered uncertain and preliminary.

## Introduction and Aim

Patient trust is based on their perception of health care personnel’s competence and interest in the patients’ well-being. There is always a certain element of risk involved in being treated for medical conditions. According to the World Health Organization (WHO), health care-related adverse events can be described as “iatrogenesis” [18]; such events include hospital-acquired infections, severe side effects of medications, or great pain due to operations. Such events occur for 1 out of every 10 patients who are receiving hospital care in high-income countries [35]. Additionally, skepticism toward vaccination programs should be seen from a perspective of trust, distrust, and risk, as demonstrated in a qualitative study by Helps et al. [13]. Medical treatment involves a certain risk, and this risk is by no means a marginal issue.

To cope with this situation of potential risk, patients need to trust health care personnel. Empirical research suggests that between patients’ knowledge about their medical condition, the treatment they receive, and their trust in health care personnel are related. Researchers and practitioners should strive to develop an understanding of this dynamic, and our paper is an attempt to contribute to this issue.

In this article, we aim to discuss the following issue:

**How can the relationship between patients’ knowledge and their trust in health care personnel be explained?**

We explore the question with a theoretical, empirically informed interdisciplinary discussion. We apply John Dewey’s (1859–1952) philosophy of education, the sociological theory of Niklas Luhmann (1927–1998), and recent empirical research in health care. The discussion is situated in a context of high modernity, as described by Anthony Giddens (1938-).

## Mythological Input

In the mythology of the Bible, we find angels. They typically have a divine character, carrying powers far exceeding those of humans, and usually, they do their deeds on behalf of God. However, they are not necessarily ‘good’ in the sense of comforting and helping people. In many of the texts, especially from the Old Testament, they are often described as intimidating beings, and people usually react with fear when they encounter them.

This is also the case in the Biblical Christmas story: The angels came to the shepherds at night to tell them that the son of God was being born, and their first reaction was that ‘they were sore afraid’. However, the angels calmed them down, assuring them that they were there to announce ‘good tidings of great joy’ (Gospel of Luke, Chap. 2 according to The King James Bible) [38]^1^.

Health care personnel are sometimes described as angels. In most cases, this is meant as a great and well-deserved compliment. However, the short introduction above reminds us of other traits of health care personnel. When a patient interacts with health care personnel, it is a meeting that hopefully is beneficial for the patient. Patients normally hope for some sort of treatment, perhaps resulting in improvement or healing, as well as some advice on how to cope with their illness or some soothing words. However, like the shepherds confronting the angels, patients are often afraid. They cannot be certain that the meeting with the health care personnel will end well. Patients may be maltreated, they may receive false or incomprehensible information, or their anxieties may be heightened by a careless word or some harsh intervention.

On the one hand, health care personnel possess unusual abilities—at least one competence that other people do not typically have: they have the knowledge, professional networks, and access to equipment and buildings that enable them to do extraordinary acts, giving them insights into other people’s bodies, minds, and lives. Furthermore, and probably most importantly, they can save lives, heal diseases, and comfort individuals when they are in great pain or deeply worried.

On the other hand, like the mythological figures of angels, there is a Janus face to the great powers of health care personnel. Their actions can potentially hurt others, they can be unsuccessful in their efforts to save lives, their skills can fail to meet patients’ expectations, and even their agenda might not be in the best interest of their patients.

## Patient Trust

Trust is often described as a necessity for a successful patient-doctor encounter, but yet, the concept of trust is often vaguely described. It can even be argued that trust is *not* needed in all such treatment, Holland and Stocks argue that *reliance* – and not trust – will in most cases be the most appropriate attitude for most medical encounters [17].

In this paper, we emphasize the action approach to trust, seeing it as the willingness to perform acts in interaction with others – or to allow others to perform a given act or set of acts. Trust-based actions are taken despite the presence of risk, uncertainty, or a lack of knowledge. From an action perspective, trust involves more or less consciously leaving something of a given value to the control of others [12, 21]. Harald Grimen (1955–2011) argued that discussing what trust *is* should not be emphasized too much, but one should rather pay attention to what trustors and trustees *do*.

Furthermore, there is a relationship between knowledge and trust. Luhmann referred to Georg Simmel (1858–1918) when he stated that trust is “a blending of knowledge and ignorance” [21]. We will return to the concept of knowledge later in this paper, but in short, we suggest that knowledge is inner representations that are generally valid, whereas “ignorance” is what is not or cannot be known. In a health care setting, the patient may be aware of his or her situation and have an idea of the doctors’/nurses’ skills but be “ignorant” of whether the care they provide will be effective. Trust is, therefore, necessary to make use of their services. Patients’ trust in health care personnel in a society of high modernity demands a willingness to overcome what is not comprehensible. Giddens emphasizes that trust in our high modernity society entails a “‘leap into faith’ which brackets ignorance or lack of information” [11].

Therefore, knowledge is crucial to forming trust, but trust also involves more than the knowledge attained in the past, and Simmel suggested two slightly different kinds of trust. One kind is primarily based on former experiences such as natural phenomena and mechanisms, and “this kind of trust is only a weak form of inductive knowledge”; another kind of trust is trust in persons and social systems, “but in the case of credit, of trust in someone, there is an additional element which is hard to describe: it is most clearly embodied in religious faith” [29]. Luhmann applied this notion of knowledge related to trust and argued that trust does more than just rely on information; it ‘exceeds information’.

[Trust] rests on the trustor being already au fait with certain general features, being already informed, even if incompletely and unreliably [21]. Hence, believing in the ‘mystery’ of trust may be a necessity on the problem of coping with the future. As earlier noted, trust involves performing an action or allowing others to act on the individual’s behalf if it is felt “secure enough” [34].

Therefore, we assume that trust is based not only on information but also on belief. Trust is related to the future. If the individual has a sense of being surrounded by a harmless environment and persons with good intentions, there are fewer things to worry about in the following seconds, days, or years. Otherwise, if a patient is uncertain about the goodwill or competence of the nurse, doctor, or psychiatrist, he or she will take precautions. Precautions in such settings may include asking for more information, asking for a second opinion and other acts that can increase positive expectations of the near and/or distant future.

An empirical finding that is relevant for the ‘future’ aspect of trust is reported in the logistic regression analysis of a survey on trust in surgical doctors [4]; the results show that low mental well-being is related to lower trust in personnel. An explanation for this finding may be that low mental well-being is related to the patients’ future outlook because the depressive state entails few and normally negative expectations of the future; here, depression is linked to low trust in others [28, 26].

As already described, the future is *unknown* and is thus a matter of ignorance. On the one hand, everyone must relate to the future, but no one can claim to *know* anything about it: “In actuality, there is less information available than would be required to give assurance of success”, as Luhmann puts it [21]. The concept of trust is such an attempt to solve the problem of uncertainty of the future as described by Eminem: “The truth is you don’t know what is going to happen tomorrow. Life is a crazy ride, and nothing is guaranteed”. Despite ignorance, individuals are more or less conscious that nothing is guaranteed and that they must act. Søren Kierkegaard famously stated that “life can only be understood backward; but it must be lived forwards” [19]. Therefore, there is no certainty about the future, but the future must be dealt with.

In the empirical literature, we can find examples of patients placing trust in health care personnel based on their assumption that the personnel do not have motives that conflict with the patients’ best interest. This trust is demonstrated in a qualitative study on trust in surgeons by Henderson and Chien and in a mixed methods study on decision-making for low-risk papillary thyroid cancer patients by Sawka et al. [14, 27]. To a certain extent, trust in health care personnel may be based on a lack of distrust. Most patients are aware that treatment can potentially be harmful or unsuccessful, and they must therefore exclude these as reasons to distrust the personnel.

The complexity and uncertainty of the future must be addressed, according to Luhmann [21]. What may happen in the future, near or far, is unknown, so individuals have no other choice but to trust the ground on which they walk, the air they breathe, and the people who surround them. If they find elements such as these to be uncertain, their possibilities for action are reduced, and they must spend resources to reduce risk. This complexity must be reduced to avoid paralysis, and in Luhmann’s perspective, trust is one of the ways to achieve this reduction of complexity. In a health care setting, patients’ trust in the health care personnel also reduces complexity: the more a patient trusts the nurses and doctors, the less complex the situation will feel, meaning that he or she will be in a high-trust situation and feel that everything can be handed over to the medical staff.

Luhmann pointed out that the future is a burden on a person’s ability to represent things to him or herself. The individual must live in the present, with the experiences of the past, and with the future in mind – and the future can seem overly complex: “They must, therefore, prune the future so as to measure up with the present, that is, to reduce complexity” [21]. Because the experiences of the past cannot cover all possible future events, this must be resolved somehow, and trust is one solution to cope with the complexity of the future.

As Luhmann described, trust is also enabling action. The concept of reduced complexity contributes to this matter, as a precondition for action is the need to reduce complexity, and trust is one of the factors that reduces complexity, a complexity “which enters the world in consequence of the freedom of other people” [21].

The empirical findings underline the forward and action dimensions of both trust and knowledge. In the qualitative study by Herwig et al. [15], patients reported that their trust in the doctor assures them of being able to assess their health situation in the future. “I would notice when something is about to happen”, as one of them stated [15]. Sawka et al. [27] found that trust in health care personnel was one of the determining factors in patients’ decision-making; again, the action and future-oriented nature of trust seems evident.

The future is not a matter of certainty but rather a matter of expectations, as Grimen [12] claimed. A ‘trustor’ will, according to his argument, leave something of value in the hands of the trustee and will not expect any harm. Grimen argued that these expectations must be seen in context; for example, in countries with great corruption, the population will not trust the legal systems, political institutions, companies, and other organizations. Indeed, past experiences determine trust, which points to the expectations of others and the concept of expectations for the future [12].

Trust is certainly based on past experiences, but unlike, for instance, satisfaction, the concept of trust has a clear future-oriented dimension. In the prelude text of this paper, the shepherds reacted with fear when they surprisingly were visited by angels, which may be based on their expectations. According to the mythology of angels, these beings can be sent to do harm. These negative or mixed expectations make a poor basis for trust in the angels. However, in this case, the expectations could be modified. The angels’ effort to explain their business that night was successful; the shepherds believed their message and even ended up enjoying a divine choral performance.

It should indeed be noted that even if trust in health care personnel in a certain sense can “replace” patients’ knowledge, it is an important ethically – and legal – matter to keep patients well informed. This is perhaps especially important and demanding in mental health care [1]. Patient autonomy is of great concern for numerous reasons, and as such “blind trust” is in no way a desirable objective.

## Patient Actions

Awareness of risk entails that a patient cannot voluntarily be treated by health care personnel unless he or she has more trust than distrust in the personnel. There may be a far greater risk not trusting health care professionals than trusting them since the alternative is no treatment or treatment by nonprofessionals. Hence, there is risk, trust, and action are connected.

Action and trust are related inasmuch as (1) action can be trust based, (2) action is enabled by a reduction in complexity, and (3) action is related to knowledge, and knowledge is a basis for trust.

First, the performance of actions, both cooperative and even individual actions, can be based on trust. Simmel described two approaches to trust, one based on “calculable probability” and the other approach, which Simmel stated is hard to describe, “is clearly embodied in religious faith” [29]. Skirbekk states that patients’ trust in health care personnel is rarely based on knowledge of the outcome of a treatment or concrete risk calculations [30]. As these actions cannot be based on calculation alone, they must be trust based. These two approaches do not exclude each other: trust-based actions will always include an element of calculation, and perhaps no actions can be based merely on calculated probability. However, in health care settings, most patient actions such as choosing to be treated and sharing personal information will include both calculation and trust. Both calculative-based and trust-based actions are related to time and to the future. A person who plans for an action has, as Luhmann argues, no information about the future. Furthermore, trust-based actions rely on how others act. For instance, if a doctor recommends that a patient use a certain medication, the patient’s willingness to adhere to the advice will be based upon his or her trust in the doctor (and trust in a great variety of other institutions and persons).

A second aspect of trust and action is the reduction of complexity. As noted above, Luhmann argues that trust enables the individual to reduce complexity, and this reduced complexity enables action [21]. By trusting the doctor, the patient may exclude or at least reduce his or her worries about the negative side effects of the treatment. The reduction of complexity is at the core of trust, Luhmann suggests. When a person seeks health care services, he or she can potentially experience a vast number of issues. The person can be rejected, can be given harmful treatment, or can be treated correctly but with no compassion or respect; furthermore, personal information may be disclosed to irrelevant people, In commercial health care, the patient may be billed far more than he or she can afford, and so on. To act in this setting of complexity, the individual simply must trust.

Finally, action is related to knowledge in the sense that through action, the individual gains experience, and experience is a basis for knowledge. Knowledge opens up room for future actions and experiences [6]. For instance, only an individual who has knowledge about how to seek health care can do so. In the words of Johan Dewey, experience is a fundamental category of knowledge. It should be noted that “experience” in this sense is not in opposition to “theory” or symbolic communication such as reading. Experience is simply an expression of the inner processes of the individual when interacting with the external world, and it involves intellectual, emotional, moral, and social aspects.

This concept of experience may be helpful in understanding how the individual forms knowledge in health care and how knowledge is related to action. When a patient performs, for instance, the act of entering a surgical department with which he or she is not familiar, he or she forms experience. This experience may include feeling welcome or not (emotional), trying to obtain an overview of the physical environment (cognitive), and making moral or at least normative assessments of whether this is a good place to be.

## Patient Knowledge

As noted earlier, empirical evidence suggests a relationship between patients’ knowledge and their trust in health care personnel. We will try to clarify a view on the knowledge that may contribute to an understanding of this relationship.

In this context, knowledge should be seen as action-oriented, as we discuss patients’ knowledge in situations where they are in the process of being treated by health care personnel. Knowledge can have the function of helping individuals cope with a situation. Ruth Anna Putnam emphasizes Dewey’s position on the issue, suggesting that knowing is firmly related to action. This involves an approach replacing the traditional spectator theory of knowledge with a theory that regards the knower of the world as an agent in that world [25]. Knowledge is a result of experience and inquiry; this entails that the rejection of any knowledge need not be certain in an absolute sense but rather have what Dewey describes as “warranted assertibility” [8, 25]. Therefore, for something to be claimed or “asserted” by an individual, she or he must find the claim warranted to some extent – there must be a reason to find the claim valid. These assertions are warranted through ongoing, self-corrected processes of enquiry [25].

Both knowledge and trust are related to the past. However, Dewey and other philosophers within the pragmatism tradition tend to explain such a phenomenon from other, less past-dwelling perspectives. Dewey’s concept of continuity also involves a future and action orientation and then action to cope with problems [6]. Anna Ruth Putnam quotes Dewey, saying that “thinking would not exist, and hence knowledge would not be found, in a world which presented no troubles” [25]; Putnam claims that this position entails that only by acting upon an idea can it be found to be adequate or not [25].

Dewey described the future-orienting function by comparing the development of knowledge to an adventurer’s work. Adventurers explore new land and shores, and during their journey, they usually draw maps. These maps are not merely a documentation of their experiences (looking back) but come into use for their own and others’ subsequent journeys; as such, they form a basis for new experiences or “further growth”, as Dewey often puts it (looking forward) [6]. Hence, knowledge is future related.

Some of the empirical findings mentioned shed light on the matter of future orientation. One example is the statements made in Conradsen et al. [3] and in Henderson and Chien [14] by the researchers’ informants regarding how patient education provided them with a sense of safety and made them more confident of their future outlook during and after their operation: “When my doctor told me that I needed to take this operation because it could totally resolve the problem – I made up my mind to take this operation” [14].

John Dewey’s concept of *continuity* entails seeing experiences and impressions as *interrelated* and concerning the *end goals* of knowledge. Knowledge of what has taken place in the past, expectations of the future, and action, together with emotional, moral, physical, and creative aspects, should all be seen as part of how learning takes place. From this perspective, one may find knowledge not to be so much a noun as a verb. Knowledge is an action rather than an entity; it is dynamic and constantly changing.

Lars Løvlie explains the concept of continuity as being about seeing phenomena as interrelated and about the end goals of knowledge. This concept includes mental, physical, thinking, and emotions [22]. Hence, the continuity concept entails that the formation of knowledge has a future orientation. Dewey described subject matter, or curriculum, as “a guide to future experiences” [7]. Hence, past—and present—experiences are used to cope with the future. This combination of past and future perspectives is a part of the concept of continuity. What an individual has done, seen, read, felt and reflected upon in the past and the present situation form a ‘guide’ for the future in as far as the way that this knowledge that is formed shapes opportunities for the future. For instance, if someone learns to read, he or she is not limited by what she or he has been trained to do; instead, reading skills open opportunities for future experiences [6].

This future perspective entails that not only are opportunities being formed but expectations of the future are also taking shape. Thus, a person who receives patient education as part of the preparations for an operation will be enabled to establish various understandings of what may come. He or she will try to establish expectations about whether the treatment will be painful, if he or she will ever recover fully, how long the hospital stay will be, etc. Patient education ahead of operations will usually include information on what to expect during the operation, in the postoperative phase, and in the long term [24]. Hence, knowledge in a treatment process should be seen from a functional and future-oriented perspective, which is captured by Dewey’s concept of continuity.

For instance, a person with epilepsy can learn breathing techniques and other cognitive skills to cope with epileptic seizures. If the individual finds that he or she can successfully deploy these skills, it may enable him or her to do activities in the future that he or she would otherwise feel to be too risky and to avoid situations that trigger such seizures. A more abstract and generalized skill is related to future actions, such as having knowledge of the health care and welfare system, which helps the individual feel competent in communicating with health care personnel. Hence, having this knowledge can modify expectations of the future and enable action.

The shepherds from the prelude of the paper used their internalized knowledge – reliable or not – to interpret and navigate with their encounter with the angels. The information they had about such beings was mixed, and they had good reasons to react with fear. We can assume that the most relevant action they considered was to flee the scene. As the story says, new information provided by one of the angels enabled them to trust the group of angels, which resulted in the shepherds acting by staying and then discussing their further action: “Let us now go even unto Bethlehem, and see this thing which is come to pass, which the Lord hath made known unto us” [38].

Action is, as Dewey sees it, a necessity for human life. The thinking process is not in principle different between science and everyday thinking. Thus, Dewey claimed the following:

Observation passes into the development of hypothesis; deductive methods pass into use in the description of the particular; inference passes into action, all with no sense of difficulty save those found in the particular task in question. The fundamental assumption is *continuity*. [5].

In a health care setting, knowledge is perhaps especially closely related to action, and health care personnel will assess medical knowledge and patient knowledge in terms of action. Knowledge of how medications work and their side effects is only of interest if it can be utilized in the treatment of patients. Likewise, patients will, as empirical studies demonstrate, find information that is relevant for future actions to be of great importance [3, 16].  It is vital to note that mistrust or trust deficiencies are not necessarily due to a lack of knowledge on the part of the patient. Failing to listen to patients and not treating them as a source of knowledge will undermine trust, as several empirical works show. Miranda Fricker describes this unwarranted distrust as “testimonial injustice”. This concept can be used to describe a situation where the patient is not considered by the professionals to be a reliable source of information because of identity prejudice. Fricker points out that this specific kind of misjudgment is “discriminatory, but ingenuous” [9]; it happens without deliberate intention to treat the speaker in an unjust manner. For a patient to establish and maintain trust in health care professionals, he or she should be felt listened to and respected, regardless of their social identity. Several empirical studies conclude that being taken seriously and listened to is fundamental for trusting the professionals [3, 28, 31].

Based on the discussions in these sections, we argue that knowledge is forward- and action-oriented by nature. Hence, it can modify trust in health care personnel because trust is also teleological, being forward- and action-oriented. We suggest that this explains the empirical relationship between knowledge about treatment, posttreatment situation, etc., and trust in health care personnel.

Patients will put less trust in health care personnel if they do not feel that they provide reliable or sufficient information about what is likely to happen in the future. In addition, patients who feel well informed tend to trust such personnel more. Knowledge enables future outlook and action, as does trust.

## The Patient in High Modernity

As trust and knowledge are constructed in social environments, we should ground this discussion in a social context. The context forms specific preconditions for the process of forming knowledge and trust. In most of the Western world, we can describe the context as dominated by high modernity, which entails that knowledge can be seen as temporary and unstable and furthermore, the functionality of the systems as largely inaccessible. For this reason, citizens must be willing to ‘bracket knowledge’ and trust the systems to make use of them [11].

One particular trait of high modern society is what Anthony Giddens denoted as *abstract systems*. Giddens described these as comprising two parts: *expert systems* and *symbolic tokens*. The expert systems are the most relevant to our issues^2^. There are a great number of expert systems in a high modern society that serve different functions. This differentiation in high modern society involves complex organizations, advanced technology, and multiple legal rules and systems [32]. For individual citizens, it is impossible to comprehend how these systems work in great detail.

The expert systems are almost impossible to understand and are inaccessible to the public, even to the persons directly involved in them. Examples of these systems are banking systems, public administrations, all kinds of commercial businesses, sports systems, and health care. Newer such systems are the digital industry and, indeed, social media. Citizens use these systems based on trust. There is no way the public can possibly know and assure themselves that these systems are reliable. The lack of knowledge about the systems means that citizens’ use of the systems is based on trust, and these systems thus depend on trust. This is certainly the case in citizens’ use of health care services and their cooperation with health care personnel.

There are quite a few examples from recent history of when systems such as these have lost the public’s trust, and when this happens, an effect on everyday life is immanent: The financial crisis that started in 2007 had a great impact on millions of people who lost both jobs and money. Other examples are resistance to vaccines, which became relevant not least during the outbreak of swine flu in 2009, and conspiratorial skepticism toward medical advice and vaccines during the COVID-19 pandemic; these are all examples of the consequences of public distrust toward expert systems [23].

Giddens describes this aspect of trust in abstract systems of high modernity as a “willingness to bracket ignorance and lack of information” [11]. This willingness to bracket ignorance in health care partly arises through patients’ encounters with the personnel who act as what he calls *access points* [10] or what Michal Lipsky [20] describes as *street-level bureaucrats*; they represent the system but also use their skills and discretion in concrete patient–personnel encounters^3^. Street-level bureaucrats are not merely points of contact between the system and the users; they engage in face-to-face interactions with the public, make decisions, make compromises, and become emotionally involved, happy, worried, distressed, and so on.

Trust in professionals in high modernity can be based on two approaches, often combined: an evidence-oriented approach, where the professionals must be accountable for their work in terms of using methods that are scientifically, empirically sound, but also based on the professional’s trustworthiness and judgement. Allison Brady discusses trust in teachers in a high modern society that is often dominated by demands for accountability. She argues that trust should not merely be based on measurement and “necessary simplifications of practices”, but on parrhesia; “Contrary to the idea that trust can only be attained should we be able to provide clear and distinct evidence that ‘proves’ that what we say is accurate, this is a trust that is instead concerned with sincerity” [2]. But discretion in complex “wicked problems” can also be coped with in a more rigorous manner. Erin Taylor argues that a procedural approach that entails a systematic investigation may provide both more unified justification for medical practice and ethical guidance for many such problems [33].

Bernardo Zacka discusses the role of discretion for street level bureaucrats and argues that (1) the complexity of their work makes a narrow understanding of their role and performance little useful, and therefore a compliance model of bureaucracy responsibility would be a “mistake”. (2) Furthermore, Zacka finds that “even if it were possible to engage in institutional reform, we would still have good reason to preserve a substantial margin of discretion at the front lines of the state” [37].

The functions of these personnel include not just providing services but also *not* providing services; they have a gate-keeping responsibility. To ensure a reasonably just distribution of the goods – and burdens – they have in their jurisdiction, they take their regulating role as the core of their professionalism [20]. A nurse must accordingly to make sure that he or she takes as good care of *all* of his or her patients as possible, and the surgeon is obliged to strive to provide the best treatment possible for all of his or her patients, whether or not he or she is perceived as nice by the grateful patients. As in the mythological prelude of this paper, the public tends to have mixed emotions related to street-level bureaucrats. Because they act discretely, there will be an element of personal judgment and therefore unpredictability in the services they provide.

The role of the street-level bureaucrats can be understood as representing the authorities, but they are also in direct contact with the part of the population for which the policy is to be exercised, relating directly to the public’s reactions, everyday life, and impact of their work. For health care personnel and other street-level bureaucrats, discretion in their professional practice is central. They must safeguard both the interests of society and the individual user’s best interest, and in many cases, there can be conflicting considerations.

Henderson and Chien [14] found in their study that patients who underwent an operation used varied strategies to reduce this complexity; they either sought reduced complexity by trust in the information about the treatment as such, or they felt reduced complexity by trusting the personnel, their agenda, and their skills. We can assume that most patients will make both of these assessments regarding the effectiveness and safety of the *treatment* and the attributes of the *personnel*. A study on whether patients would trust artificial intelligence (AI) in medicine more than live doctors found that certain parts of the population and within certain fields of medicine, individuals are more trustful toward AI than doctors. More highly educated males with Western backgrounds and those not admitted to the hospital in the past 12 months reported the most positive feelings toward AI. However, the general view of the 2411 respondents in the study toward the use of AI for treatment was more negative [36]. These studies demonstrate that trust in personnel is complex and should be studied in specific contexts. The study also indicates that discretion is vital for most patients to form trust in the patient-health care personnel relationship, but technology modifies the basis of this relationship.

The previously described principle of continuity entails that patients in high modernity have experience with expert systems and street-level bureaucrats (personnel), and these experiences form knowledge. The ‘curriculum’ of these experiences enables—or disables—trust, and the outcome of this knowledge and trust is a ‘guide to future experiences’ [6].

To describe the nature of the trust relationship between the street-level bureaucrat and the user in a health care setting, the concept *mandates of trust*, as described by Skirbekk et al., may be useful. A service user, for example a hospital patient, must give the health care personnel this mandate in order to delegate to such personnel the authority to act in the best interests of the patient. This mandate can be of a narrow nature, allowing only a limited set of actions to be performed, such as a slightly invasive surgical procedure. In other situations, the mandate can be wider, such as in the communication between a patient and a general practitioner about complex and long-lasting issues [31].

Understanding how patients relate to health care professionals and see them as access points to expert systems of high modernity could be a fruitful way to analyze the relationship between knowledge and trust in health care. Trust, as a social phenomenon, should be interpreted with the context in mind, and we have tried to clarify how the perspectives of Luhmann, Giddens, Lipsky, and Skirbekk can be useful in understanding trust in health care in high modernity.

## Conclusions

We started by asking how the relationship between patients’ knowledge and their trust in health care personnel can be explained and have discussed this matter by highlighting the contributions of John Dewey and his concept of continuity, combined with the trust theory concepts of Niklas Luhmann and others. In the context of high modernity, expert systems appear to be rather difficult to comprehend and rely on the trust of the public. The role of health care personnel is therefore essential, as they must act discretely to establish the necessary trust.

The main conclusion is that the relationship between patients’ knowledge and their trust in health care personnel can be explained by a future and action orientation. John Dewey’s concept of continuity seems applicable in describing this relationship between knowledge, trust, and future and action orientations. The context of high modernity entails specific possibilities and challenges in the patient-personnel encounter in terms of how knowledge is experienced as complex and uncertain. This setting also demands that health care personnel act discretely and represent systems that require a great amount of trust from the public.

The concept of trust entails a future orientation, and as Dewey emphasizes, knowledge enables future experiences, trust points the way forward, and if a patient can trust health care professionals, doing so will provide a basis for treatment and communication. There is risk involved in this, as trust relies on “ignorance”, and one must accept not actually knowing how others will act and whether the professionals’ advice will actually be helpful to the patients’ health.

We find it meaningful to use the continuity concept for discussing trust issues. It may be helpful to use this concept in empirical studies because it helps explain why knowledge can or cannot enable trust and distrust. Such discussion may provide a conceptual approach to examining trust by providing a framework of learning theory that involves an interest in how patients strive to cope with their own future and in how to interact with health care personnel in a highly modern society.

## Footnotes

1 This observation of the paradoxical nature of angels in the Judeo-Christian tradition is borrowed from the novel *A Time to Every Purpose Under Heaven* by Karl Ove Knausgård.

2 Symbolic tokens are entities such as money and other items that have no or little value but are given importance by a prescribed value accepted by society.

3 Giddens’ concept of “access point” refers to both personal and “faceless commitments”, whereas the Lipsky expression “street level bureaucrat” indicates personnel who face the public, such as policemen, social workers, teachers, doctors and nurses.
